# The innate immune response in human tuberculosis

**DOI:** 10.1111/cmi.12480

**Published:** 2015-07-28

**Authors:** Thomas R. Lerner, Sophie Borel, Maximiliano G. Gutierrez

**Affiliations:** ^1^ Mill Hill LaboratoryThe Francis Crick InstituteLondonUK

## Abstract

*M* *ycobacterium tuberculosis* (Mtb) infection can be cleared by the innate immune system before the initiation of an adaptive immune response. This innate protection requires a variety of robust cell autonomous responses from many different host immune cell types. However, Mtb has evolved strategies to circumvent some of these defences. In this mini‐review, we discuss these host–pathogen interactions with a focus on studies performed in human cells and/or supported by human genetics studies (such as genome‐wide association studies).

## Introduction

*Mycobacterium tuberculosis* (Mtb) is particularly effective at subverting many of the host immune defences, and this is one reason why it is such a successful human pathogen that has been particularly hard to eradicate. The outcome of infection by Mtb and therefore the clinical manifestation of tuberculosis (TB) depend on many combined factors, such as host genetics, bacterial genetics (virulence factors), the health and nutritional status of the host and whether there has been any prior exposure/immunity and vaccination history. Around half of individuals exposed to Mtb do not exhibit a positive tuberculin skin test (Morrison *et al*., 2008), indicating that infection did not occur after exposure or that there has been no Th1‐type adaptive immune response (forming characteristic granulomas), indicating that there may have been an ‘early clearance’ of Mtb by the innate immune system (Verrall *et al*., 2014). All the information discussed in this review has been gained from studies using human cells or patients, because it has been shown that there are often differences with animal models that can affect the outcome of TB infection (Fortin *et al*., 2007). Although human primary cells are relevant models for studying human TB, there are difficulties associated with their use such as donor variability and genetic manipulation. Therefore, human macrophage‐like cell lines are also an important tool, as long as the data obtained using them are discussed with their aberrant nature in mind. Moreover, in both cell lines and primary cells, different aspects need to be considered such as multiple differentiation protocols that impairs useful comparisons across different laboratories and suboptimal culture conditions of the physiological environment (Vogt and Nathan, 2011).

First, we discuss the different cell types important for innate immunity against Mtb. Then, we discuss the mechanisms these cells use to clear the infection and the Mtb effectors that subvert the host defences.

## Cells involved in the innate immune response to TB in humans

### Innate defences to Mtb in the airways: the respiratory mucosa

**Figure 1:**
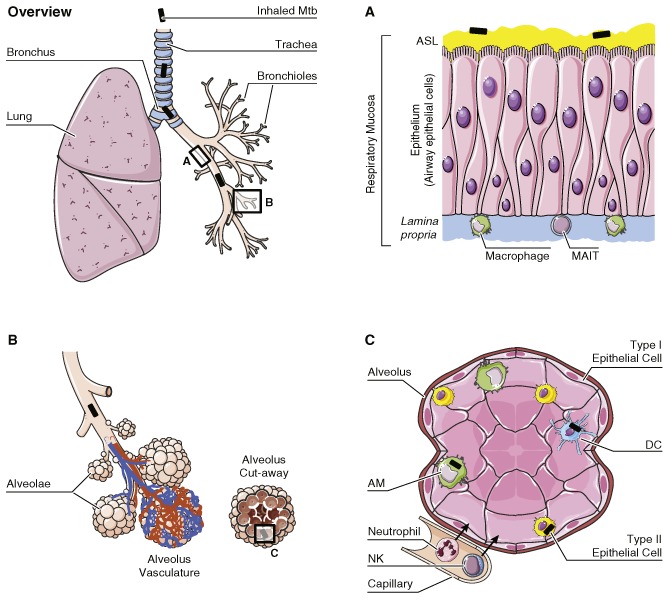
Cells involved in the human innate immune response to tuberculosis. Upon inhalation into the lung, Mtb (black rod) travels along the trachea, bronchus and bronchioles to the alveoli. Lining the airway is the respiratory mucosa (A). This consists of a layer of AECs that provide a tight barrier to prevent Mtb from invading the tissue and they have many receptors to detect Mtb. AECs control the composition of ASL, a substance containing mucus, anti‐microbial peptides, antibodies and cytokines/chemokines. The lamina propria supports the epithelium and also contains immune cells such as macrophages and MAIT that respond to infection. Mtb eventually reach the alveolae (B), which are surrounded by a network of capillaries to facilitate gas exchange. The alveolus (C) is structurally formed from type I epithelial cells, and type II epithelial cells are often found at the cell junctions. Type II cells secrete a variety of anti‐microbial substances including pulmonary surfactant. AMs and DCs are the primary resident defenders of the alveolus. They are effective phagocytes and have a range of intrinsic anti‐microbial capacities. In addition, neutrophils and NKs are recruited from the surrounding capillaries to bolster the host defence. Cells are not drawn to scale.

Mtb is inhaled through the nose and mouth and passes along the trachea, bronchus, bronchioles and eventually to the alveoli in the lung (Fig. 1). Along the airway is the respiratory mucosa (Fig. 1A) that forms the first line of defence against Mtb (Middleton *et al*., 2002). It consists of (i) the epithelium, a layer of airway epithelial cells (AECs) forming a barrier that prevents invasion; (ii) the lamina propria, a layer of connective tissue and immune cells, including lymphocytes and macrophages; and (iii) a coating of a highly complex substance known as airway surface liquid (ASL), which contains mucus, immunoglobulin A and an array of other innate immune factors on the luminal surface. Also located in prime positions along the airways to encounter Mtb are bronchial‐ or nasal‐associated lymphoid tissues that are crucial for Mtb antigen sampling (Lugton, 1999).

AECs can recognize pathogen‐associated molecular patterns (PAMPs) present on Mtb surfaces as they constitutively express pattern recognition receptors such as Toll‐like receptors, Dectin‐1, C‐type lectin receptors (CLRs), nucleotide‐binding oligomerization domain‐containing protein 2 (NOD2), dendritic cell (DC)‐specific intercellular adhesion molecule‐3‐grabbing non‐integrin and mannose receptor (reviewed in Li *et al*., 2012). These receptors have been implicated in Mtb infection in human AECs and mediate the production of cytokines and effector molecules to mount an effective immune response. AECs play a role as immune sentinels after exposure to Mtb by presenting antigen to mucosal‐associated invariant T cells (MAITs) (Harriff *et al*., 2014) and stimulating them to produce interferon (IFN)‐γ, tumour necrosis factor (TNF)‐α and granzyme, factors that may contribute to Mtb clearance. MAIT cells rapidly respond to infection, providing an early IFN‐γ boost to activate macrophages (Gold *et al*., 2010). Crucially, AECs control the composition of ASL. ASL contains anti‐microbial peptides that have been implicated in Mtb resistance, such as β‐defensin 2 (Rivas‐Santiago *et al*., 2005), cathelicidin (LL‐37) (Rivas‐Santiago *et al*., 2008) and hepcidin (Sow *et al*., 2011), as well as a variety of cytokines and chemokines that are secreted by AECs to recruit and activate phagocytes (reviewed in Li *et al*., 2012).

### Innate defences to Mtb in the airways: the alveoli

Mtb that successfully passes through the upper airways will be delivered to the alveoli (Fig. 1B andC). The alveoli consists of a thin lining of type I and II epithelial cells as well as other immune cells such as alveolar macrophages (AMs), DCs and neutrophils. Type I epithelial cells form the walls of the alveolus, and these cells are primarily involved with gas exchange. Whether they can be infected by Mtb remains to be seen. In contrast, infection of type II epithelial cells by Mtb has been extensively studied *in vitro*, and Mtb DNA has been detected within these cells in post‐mortem studies (Hernández‐Pando *et al*., 2000). Similar to AECs, these cells produce anti‐microbial molecules (Rivas‐Santiago *et al*., 2005). Additionally, type II cells produce and secrete pulmonary surfactant, hydrolytic enzymes and hydrolases in the extracellular lining of the lung. Surfactant proteins (members of the collectin family) bind to Mtb, causing agglutination (Ferguson *et al*., 1999) and enhanced phagocytosis by macrophages (Gaynor *et al*., 1995). Secreted hydrolases can alter the cell wall of Mtb and affect interactions with macrophages and host immune responses (Arcos *et al*., 2011).

### Innate defences to Mtb in the airways: resident defenders

There are relatively few AMs per alveolus (around 10), but they live for around 3 months in humans (Thomas *et al*., 1976). AMs have a whole range of cell autonomous anti‐microbial mechanisms at their disposal (see below). On the other hand, evolution has equipped Mtb with the capability to evade and/or tolerate some of these anti‐microbial mechanisms (Table 1), and the outcome of the initial battle depends on the intrinsic microbicidal capacity of the host cell and the virulence factors of the ingested Mtb. If the host cell kills Mtb, then the infection is controlled, but if this response is ineffective, Mtb will replicate in this niche and the infection will spread.

**Table 1:** Mtb virulence factors counteracting the innate immune response

Mtb effectors	Action	Mechanism	Cell type	References
Intracellular trafficking and localization				
ESAT‐6	Translocation into the cytosol	ESAT‐6 has a pore forming activity	THP‐1, DCs	van der Wel *et al*., 2007; Houben *et al*., 2012; Simeone *et al*., 2012
LAM	Inhibits phagolysosome fusion	Unknown	THP‐1/MDMs	Hmama *et al*., 2004; Kang *et al*., 2005; Welin *et al*., 2008
PtpA	Inhibits phagosome acidification	vATPase exclusion	THP‐1	Bach *et al*., 2008; Wong *et al*., 2011; Wong and Jacobs, 2011
Autophagy				
ESAT‐6	Inhibits production of IFN‐γ	Affects TCR signalling	T cells	Wang *et al*., 2009
ESX‐1 secretion system	Inhibition of autophagosomes/lysosome fusion	Unknown	DCs	Romagnoli *et al*., 2012
LAM	Blocks transcriptional activation of IFN‐γ	Unknown	U937/THP‐1	Chan *et al*., 1991
miR‐30A	Inhibition of autophagy	Unknown	THP‐1	Chen *et al*., 2015
Sulfatide	Blocks IFN‐γ or lipopolysaccharide priming	Unknown	Monocytes	Pabst *et al*., 1988
Host cell death				
ESAT‐6	Induces necrotic death	Caspase‐1‐ and cathepsin B‐independent necrosis	MDMs	Welin *et al*., 2011
ESX‐1 secretion system	Extracellular traps	Unknown	MDMs	Wong and Jacobs, 2013
CpnT	Induces necrotic death	Unknown	Jurkat T	Danilchanka *et al*., 2014
SecA2 and NuoG	Suppress apoptosis	Unknown	THP‐1	Hinchey *et al*., 2007; Velmurugan *et al*., 2007; Miller *et al*., 2010
PknE	Inhibits apoptosis	Unknown	THP‐1	Jayakumar *et al*., 2008
Rv3364c	Suppresses caspase‐1 and pyroptosis	Inhibition of cathepsin G activity	U937	Danelishvili *et al*., 2011
Reactive species and toxic metals				
Eis	Modulates ROS production	Targets JNK pathway	THP‐1	Shin *et al*., 2010
*ctpC*	Zinc detoxification	Zinc efflux	MDMs	Botella *et al*., 2011
NuoG	Neutralizes ROS and TNF‐α‐mediated host cell apoptosis	Unknown	AMs	Miller *et al*., 2010

Mtb effector abbreviations: CpnT, C‐terminal domain of the channel protein with necrosis‐inducing toxin; Eis, enhanced intracellular survival protein; LAM, lipoarabinomannan; PknE, protein kinase E; PtpA, tyrosine phosphatase. Cell type abbreviations: AMs, alveolar macrophages; DCs, dendritic cells; MDMs, monocyte‐derived macrophages.

DCs are also one of the first types of cell to encounter Mtb. They have a multitude of receptors to detect Mtb PAMPs and are highly efficient phagocytes (Henderson *et al*., 1997). After uptake of Mtb, DCs in the alveoli mature and present antigens via Major Histocompatibility Complex (MHC) class I and II to T cells in the local draining lymph node (Marino *et al*., 2004); thus, DCs are a link between the innate and adaptive immune response. However, the outcome of Mtb and DC interaction is complex and not well understood, likely to be due to variation in host genetics and bacterial virulence factors. Mtb is capable of replicating within DCs (Förtsch *et al*., 2000), and some reports show that Mtb actually manipulates DC function and impairs their ability to control infection (Hanekom *et al*., 2003). However, other studies find that DCs are beneficial to bolster the cellular immune response (Tailleux *et al*., 2003).

### Innate defences to Mtb in the airways: recruited defenders

Neutrophils are the predominant cell type infected in the airways of individuals with active TB (Eum *et al*., 2010). These professional phagocytes play a very complex and conflicting role in the pathology of TB that likely depends upon the host genetics, Mtb virulence factors and also the stage of TB disease. Reflecting this, some studies have shown that human neutrophils can either restrict (Brown *et al*., 1987) or favour (Denis, 1991) Mtb growth. Highlighting the importance of virulence factors for the outcome of infection, a study showed that in primary human neutrophils, only virulent Mtb could survive the host‐generated respiratory burst by inducing necrotic cell death (Corleis *et al*., 2012). After stimulation with Mtb, neutrophils secrete chemokines and pro‐inflammatory cytokines leading to recruitment and activation of other immune cells (Riedel and Kaufmann, 1997). Human apoptotic neutrophils infected with Mtb can be phagocytosed by Mtb‐infected macrophages; in this case, the anti‐microbial contents of neutrophil granules can directly fuse with Mtb‐containing phagosomes in macrophages, leading to improved killing (Tan *et al*., 2006). Whether human neutrophils can control intracellular Mtb or act via neutrophil extracellular traps (NETs) is still debated (see below).

Natural killer cells (NKs) are also involved in Mtb infection. These innate immune cells are recruited to the site of infection early on and play a role in amplifying the anti‐microbial defence to TB. This is via recognition of infected macrophages through receptor molecules such as NKp44, NKp46 and NKG2D (Vankayalapati *et al*., 2005). NKs can lyse infected macrophages (Vankayalapati *et al*., 2002), produce IFN‐γ to further activate macrophages and can also secrete cytokines that expand CD8^+^ T cells and NK T cell (NKTs) populations (Vankayalapati and Barnes, 2009). NKTs recognize lipid antigens presented by CD1a molecules and NKT deficiency is associated with the development of active TB (Sutherland *et al*., 2009). There are other T‐cell subsets such as γδ T cells present in the alveoli; they have been shown to recognize Mtb phosphoantigens (Ismaili *et al*., 2002) and participate in the killing of infected macrophages through cytotoxic granules.

## Cell autonomous defence mechanisms in TB

### Trafficking and localization of Mtb in human cells

The phagosome is a central mediator of both the homeostatic and microbicidal functions of macrophages. After phagocytosis, Mtb blocks phagosome acidification as well acquisition of hydrolytic enzymes and anti‐microbial peptides. Two major Mtb virulence factors are involved in the blockage of the phagosomal maturation in human cell lines: the glycolipid lipoarabinomannan (LAM) (Hmama *et al*., 2004; Kang *et al*., 2005; Welin *et al*., 2008) and the secreted tyrosine phosphatase (PtpA) (Bach *et al*., 2008; Wong *et al*., 2011; Wong and Jacobs, 2011). Mtb lacking the surface lipid trehalose dimycolate (TDM) failed to block phagosome maturation in mouse macrophages (Katti *et al*., 2008) but to date, this has not been shown in human cells. However, in humans, there is a polymorphism in the CLR for TDM CLECSF8 (MCL) that is associated with susceptibility to pulmonary TB (Wilson *et al*., 2015) and implicates TDM as an important virulence factor in human infection.

*In vitro* studies using electron microscopy (EM) and a fluorescence resonance energy transfer‐based method showed that Mtb also localizes in the cytosol in THP‐1 macrophages, primary human macrophages and primary human DCs (van der Wel *et al*., 2007; Houben *et al*., 2012; Simeone *et al*., 2012). The ESX‐1 type VII secretion system (T7SS) that is lacking from most of the non‐pathogenic mycobacterial strains (Abdallah *et al*., 2007) is required for Mtb localization in the cytosol. As part of the T7SS, ESAT‐6 protein is believed to make pores on cellular membranes (Hsu *et al*., 2003; Jonge *et al*., 2007; Wong and Jacobs, 2011). However, mechanistically, how ESAT‐6 lyses the phagosomal membrane in host cells is still unknown.

Although cytosolic localization of Mtb has been reported *in vitro*, less is known regarding the subcellular localization of Mtb in cells from patients with TB. EM studies in infected AMs isolated by bronchoalveolar lavage from infected individuals revealed that Mtb localizes primarily in membrane‐bound compartments (Russell *et al*., 2002; Mwandumba *et al*., 2004). The subcellular localization of Mtb in other cells *in vivo* and the physiological relevance of the cytosolic localization is far from clear (Harriff *et al*., 2012), but it is becoming increasingly apparent that Mtb‐infected cells are likely to have a mixed population of bacteria that are free in the cytosol or found in membrane‐bound compartments; this should be considered in future interpretation of experimental data and also in drug development.

## Autophagy in the immune response to TB

Autophagy plays a crucial role in resistance to pathogens and has been implicated as an important innate defence mechanism in controlling and eliminating Mtb (Gutierrez *et al*., 2004). Whereas many studies highlighted the role of autophagy during Mtb infection in the mouse model, less is known about the autophagic response in human cells or in patients with active TB. Mtb is able to evade autophagy by inhibiting fusion of autophagosomes with lysosomes through the ESX‐1 secretion system in primary human DCs (Romagnoli *et al*., 2012) and by expressing miR‐30A in THP‐1 macrophages (Chen *et al*., 2015). Several studies have linked vitamin D deficiency with an increased risk for susceptibility to active TB (Martineau *et al*., 2007; Wejse *et al*., 2007; Nnoaham and Clarke, 2008). The active form of vitamin D3 induces autophagy in primary human monocytes and THP‐1 macrophages via the expression of the anti‐microbial peptide cathelicidin, which activates transcription of the autophagy‐related genes encoding Beclin‐1 and ATG5 (Yuk *et al*., 2009). In primary human macrophages and THP‐1 macrophages, active vitamin D3 also induces the localization of Mtb in autophagosomes in a cathelicidin‐dependent manner (Yuk *et al*., 2009).

IFN‐γ induces autophagy in response to Mtb antigens in patients with active TB (Rovetta *et al*., 2014). In human primary macrophages, the protective effect of IFN‐γ depends on the timing of addition, concentration and magnitude of the ensuing microbial challenge (Vogt and Nathan, 2011). Mtb factors able to interfere with IFN‐γ response include ESAT‐6, which inhibits production of IFN‐γ by Mtb‐responsive primary human‐stimulated CD3^+^ T cells (Wang *et al*., 2009) and sulfatides present on the outer surface of Mtb that blocks IFN‐γ or lipopolysaccharide priming in primary human monocytes (Pabst *et al*., 1988). LAM is also able to block the transcriptional activation of IFN‐γ‐inducible genes in human macrophage‐like cell lines (Chan *et al*., 1991).

## IFN‐γ‐inducible GTPases

IFN‐γ‐induced autophagy is also required to control intracellular pathogens via members of the immunity‐related GTPase family (IRG proteins, formerly known as p47 GTPases) and by the 65 kDa guanylate binding protein family. Compared with the mouse genome (containing 23 *IRG* genes), the human genome contains only three *IRG* genes, encoding IRGC, IRGQ and IRGM, but these are not inducible by IFN‐γ. Polymorphisms in the *IRGM* gene, which is functional in humans, are associated with susceptibility to TB among African‐Americans (King *et al*., 2011), Ghanese (Intemann *et al*., 2009) and Chinese (Che *et al*., 2010) populations, providing evidence that IRG proteins contribute to the control of Mtb in humans. However, functional polymorphisms in both *IRGM* and the autophagy gene *ATG16L1* did not have a major impact on Mtb‐induced cytokine production in healthy volunteers, although a moderate effect was observed on IFN‐γ production by the *ATG16L1* T300A polymorphism (Kleinnijenhuis *et al*., 2011). IRGM and other autophagic markers such as LC3 and ATG16L1 are recruited to Mtb‐containing compartments by the activation of the innate immune receptor NOD2 in Mtb‐infected human AMs (Juárez *et al*., 2012). However, the precise mechanism by which this family of proteins control the cell autonomous response to Mtb is not known.

## Host cell death in immunity

The mode of host cell death after Mtb infection is crucial for the outcome of the disease. Mtb induces necrosis, a death modality defined by cell lysis, and inhibits apoptosis, a form of death that maintains an intact plasma membrane and that enables control of bacterial replication. Several Mtb proteins inhibit apoptosis in human cells such as the serine/threonine kinase PknE (Jayakumar *et al*., 2008) and the Rv3364c protein (Danelishvili *et al*., 2011). Moreover, the Mtb proteins SecA2 and NuoG suppress THP‐1 macrophage apoptosis (Hinchey *et al*., 2007; Velmurugan *et al*., 2007; Miller *et al*., 2010).

Once in the cytosol, Mtb induces necrosis as a strategy used by virulent bacteria to avoid innate host defence. In primary human macrophages, Mtb induces necrosis by causing mitochondrial inner membrane disruption (Chen *et al*., 2006) and inhibiting the lysosomal and Golgi‐mediated plasma membrane repair (Divangahi *et al*., 2009). In T cells, the C‐terminal domain of the channel protein with necrosis‐inducing toxin induces necrotic death (Danilchanka *et al*., 2014). Induction of necrosis is also dependent on bacterial load and a functional ESX‐1 system. Indeed, primary human macrophages infected with a high burden of ESAT‐6‐expressing Mtb undergo Caspase‐1‐ and Cathepsin B‐independent necrosis (Welin *et al*., 2011).

In neutrophils, Mtb induces NETs, which contain DNA and several biologically active cytosolic and granular proteins (Braian *et al*., 2013). The formation of NETs plays an essential function in the innate immune defence against Mtb infection by trapping mycobacteria and thereby preventing spread to other organs (Braian *et al*., 2013). *In vitro*, this mechanism has been observed in human but not in mouse macrophages infected by Mtb (Wong and Jacobs, 2013). The formation of extracellular traps by primary human macrophages during Mtb infection is inducible by IFN‐γ and requires the ESX‐1 secretion system (Wong and Jacobs, 2013).

## Reactive species and toxic metals

Another cell autonomous mechanism that controls intracellular Mtb consists of directly exposing mycobacteria to a toxic intracellular environment containing, e.g. reactive oxygen and nitrogen species (ROS and RNS) as well as toxic metals. In the murine model of *Mtb* infection, the importance of nitric oxide (NO) and RNS for the control of intracellular mycobacterial replication and disease is well established (Chan *et al*., 1992). In human macrophages, however, the role of NO is less clear. Phagocytes induce oxidative killing by production of ROS including superoxide and hydrogen peroxide. The generation of ROS requires assembly of the superoxide‐generating NADPH oxidase 2 (NOX2) complex at phagolysosomal membranes (Bylund *et al*., 2010). The role of ROS in anti‐mycobacterial immunity has been highlighted by the discovery of a mutation in the gene encoding the catalytic subunit gp91^phox^ of NOX2 linked to TB susceptibility in patients (Bustamante *et al*., 2011). Several Mtb factors counteract the production of ROS. The ‘enhanced intracellular survival’ (*eis*) gene modulates host cell ROS generation (Shin *et al*., 2010). Mtb can also neutralize NOX2‐derived ROS via a NuoG‐dependent mechanism in order to inhibit TNF‐α‐mediated host cell apoptosis in primary human AMs (Miller *et al*., 2010).

Heavy metal poisoning is emerging as a very effective cell autonomous mechanism of bacterial elimination. Transcriptional studies show that during infection of primary human macrophages, Mtb faces a burst of free zinc, which accumulates within the mycobacterial phagosome (Botella *et al*., 2011). To counteract this mechanism, Mtb up‐regulates expression of the P‐type ATPase‐encoding gene *ctpC*, which regulates the intra‐bacterial levels of Zn^2+^ through efflux of the metal ion (Botella *et al*., 2011).

## Conclusion

The host innate immune response to TB requires a variety of different host cell types to successfully protect the host from infection. Physical barriers and anti‐microbial substances are just as important as immune cells for protection. There are many different factors that influence the outcome of the initial battle between host and pathogen, including a variety of mechanisms that Mtb has evolved to subvert host defences. If Mtb is not killed by the innate immune response, it will replicate and disseminate and the host adaptive immune response will become critical for control. This review focuses solely on studies performed in humans or in human cells. The majority of the known innate mechanisms involved in Mtb infection have been studied in murine cells. Considering that there are key differences in host cell responses between humans and other animal models, one of the major challenges for the future will be to confirm the relevance of these findings in humans. Better understanding of the mechanisms involved in innate immunity in humans will enable us to develop improved treatments for TB.
